# Early non-response as a predictor of later non-response to antipsychotics in schizophrenia: a randomized trial

**DOI:** 10.1186/s12916-023-02968-7

**Published:** 2023-07-19

**Authors:** Yujun Long, Qiongqiong Wu, Ye Yang, Jingda Cai, Jingmei Xiao, Zhaoqian Liu, Yifeng Xu, Ying Chen, Manli Huang, Ruiguo Zhang, Xijia Xu, Jian Hu, Zhifen Liu, Fang Liu, Yingjun Zheng, Huaqing Meng, Zhimin Wang, Yanqing Tang, Xueqin Song, Yunchun Chen, Xueyi Wang, Tiebang Liu, Xiaoli Wu, Maosheng Fang, Chunling Wan, Jingping Zhao, Renrong Wu

**Affiliations:** 1 Department of Psychiatry, National Clinical Research Center for Mental Disorders, and National Center for Mental Disorders, The Second Xiangya Hospital of Central South University, 139# Renmin Middle RD, Changsha, 410011 Hunan China; 2 Department of Clinical Pharmacology, Xiangya Hospital, Central South University, Changsha, Hunan China; 3 Department of Psychiatry, Shanghai Mental Health Center, Shanghai Jiao Tong University School of Medicine, Shanghai, China; 4 Huaxi MR Research Center (HMRRC), Department of Radiology, West China Hospital of Sichuan University, Chengdu, Sichuan China; 5 Department of Psychiatry, The First Affiliated Hospital, Zhejiang University School of Medicine, Hangzhou, Zhejiang China; 6 Department of Psychiatry, Xijing Hospital, Air Force Military Medical University, Xi’an, Shaanxi China; 7 Department of Psychiatry, The Affiliated Brain Hospital of Nanjing Medical University, Nanjing Brain Hospital, Nanjing, Jiangsu China; 8 Department of Psychiatry, The First Affiliated Hospital of Harbin Medical University, Harbin, Heilongjiang China; 9 Department of Psychiatry, The First Hospital of Shanxi Medical University, Taiyuan, Shanxi China; 10 Department of Psychiatry, The First Affiliated Hospital of Kunming Medical University, Kunming, Yunnan China; 11 Department of Psychiatry, The Affiliated Brain Hospital of Guangzhou Medical University, Guangzhou, Guangdong China; 12 Department of Psychiatry, The First Affiliated Hospital of Chongqing Medical University, Chongqing, China; 13 The National Clinical Research Center for Mental Disorders, Beijing Anding Hospital, Capital Medical University, Beijing, China; 14 Department of Psychiatry, the First Hospital of China Medical University, Shenyang, Liaoning China; 15 Department of Psychiatry, The First Affiliated Hospital of Zhengzhou University, Zhengzhou, Henan China; 16 Department of Psychiatry, The First Affiliated Hospital of Xi’an Jiaotong University, Xi’an, Shaanxi China; 17 Department of Psychiatry, First Hospital of Hebei Medical University, Shijiazhuang, Hebei China; 18 Department of Psychiatry, Shenzhen Kangning Hospital, Shenzhen, Guangdong China; 19 Department of Psychiatry, The Third Affiliated Hospital, Sun Yat-Sen University, Guangzhou, Guangdong China; 20 Wuhan Mental Health Center, Wuhan, Hubei China; 21 Bio-X Institutes, Key Laboratory for the Genetics of Developmental and Neuropsychiatric Disorders, Ministry of Education, Shanghai Jiao Tong University, Shanghai, China

**Keywords:** Schizophrenia, Early response, Atypical antipsychotic, Prediction

## Abstract

**Background:**

It remains a challenge to predict the long-term response to antipsychotics in patients with schizophrenia who do not respond at an early stage. This study aimed to investigate the optimal predictive cut-off value for early non-response that would better predict later non-response to antipsychotics in patients with schizophrenia.

**Methods:**

This multicenter, 8-week, open-label, randomized trial was conducted at 19 psychiatric centers throughout China. All enrolled participants were assigned to olanzapine, risperidone, amisulpride, or aripiprazole monotherapy for 8 weeks. The positive and negative syndrome scale (PANSS) was evaluated at baseline, week 2, week 4, and week 8. The main outcome was the prediction of nonresponse. Nonresponse is defined as a < 20% reduction in the total scores of PANSS from baseline to endpoint. Severity ratings of mild, moderate, and severe illness corresponded to baseline PANSS total scores of 58, 75, and 95, respectively.

**Results:**

At week 2, a reduction of < 5% in the PANSS total score showed the highest total accuracy in the severe and mild schizophrenia patients (total accuracy, 75.0% and 80.8%, respectively), and patients who were treated with the risperidone and amisulpride groups (total accuracy, 82.4%, and 78.2%, respectively). A 10% decrease exhibited the best overall accuracy in the moderate schizophrenia patients (total accuracy, 84.0%), olanzapine (total accuracy, 79.2%), and aripiprazole group (total accuracy, 77.4%). At week 4, the best predictive cut-off value was < 20%, regardless of the antipsychotic or severity of illness (total accuracy ranging from 89.8 to 92.1%).

**Conclusions:**

Symptom reduction at week 2 has acceptable discrimination in predicting later non-response to antipsychotics in schizophrenia, and a more accurate predictive cut-off value should be determined according to the medication regimen and baseline illness severity. The response to treatment during the next 2 weeks after week 2 could be further assessed to determine whether there is a need to change antipsychotic medication during the first four weeks.

**Trial registration:**

This study was registered on Clinicaltrials.gov (NCT03451734).

**Supplementary Information:**

The online version contains supplementary material available at 10.1186/s12916-023-02968-7.

## Background

Antipsychotic drugs are the main treatment method for schizophrenia. However, the effectiveness of this therapy in clinical practice is unsatisfactory; 19.8–66.9% of patients with schizophrenia do not or only partially respond to antipsychotic in 4–6 weeks [1]. The response is characterized by a clinically meaningful improvement in the patient’s clinical symptoms and is estimated by a percentage reduction of the initial total score (usually ranging from 20 to 50%) on symptom rating scales like the Positive and Negative Syndrome Scale (PANSS) [2]. It typically requires a period of treatment for clinicians to assess a patient’s response to antipsychotics. Although the initial response has been considered an important determinant of long-term prognosis in first-episode psychosis, the timepoint to change antipsychotic medication, if the patients fail to have a good response, is still an unresolved clinical issue [3].

The assertions in treatment guidelines continue to be diverse and commonly lack sufficient supportive evidence. The World Federation of Societies of Biological Psychiatry (WFBSP) gave a wide range of 2–8 weeks at the recommended dose before switching medication [4]. The National Institute for Health and Care Excellence (NICE) and the British Association of Psychiatry (BAP) suggest keeping antipsychotic medication therapy at the recommended dosage for 4–6 weeks [5, 6]. Some recent studies have found early non-response to antipsychotics (insufficient improvement seen within 2–4 weeks) could predict later non-response [7–9]. A diagnostic test meta-analysis on individuals’ data by Samara et al. revealed that patients are unlikely to continue to improve if they get non-improvement by the second week of antipsychotic medication [10]. These studies, however, were not disaggregated by baseline severity status and antipsychotics.

Baseline severity of schizophrenia has been reported to influence the response to antipsychotics that patients with more severe initial symptoms tend to experience greater reductions in symptoms [11]. Some studies have indicated that initial symptom severity is a possible predictor of later changing in antipsychotics for patients with schizophrenia [12, 13]. Furthermore, the baseline severity of clinical symptoms was found to be one of the independent factors determining the specificity of early prediction of long-term nonresponse [10]. Yet, only a few studies have investigated whether early prediction of non-response varies in subgroups with different levels of severity, and these results were inconsistent. One study found that in severely ill patients (Clinical Global Impression-Severity of Illness Scale, CGI-S ≥ 5 points), non-response at week 4, but not at week 2 was predictive for later non-response (< 25% reduction in PANSS total score or CGI-S < 2 points improvement) [14]. While in another study including patients who were at least moderately ill (PANSS total score ≥ 75) at baseline, early non-response (< 20% improvement in PANSS total score from baseline) at week 2 could robustly predict subsequent lack of response (< 40% reduction in PANSS total score) [15].

Besides the baseline severities of illness, the type of antipsychotics also plays a role in predicting early nonresponse in subsequent treatment nonresponse. For instance, one study focusing on acute-phase schizophrenia with the definition of early response as a Clinical Global Impressions-Improvement Scale (CGI-I) ≤ 3 after 2 weeks of treatment found that response to risperidone at 2 weeks can effectively predict long-term treatment response, whereas olanzapine required 4 weeks [16]. Another study using the PANSS to define non-responders (< 20% reduction rate) found that a 2-week response to risperidone effectively predicted 4-week and 6-week responses in patients with acute-phase schizophrenia [17]. Similar findings were obtained in two other studies that used regression analysis, which found that patients who received olanzapine needed more than 2 or 3 weeks to predict longer-term improvement (≥ 50% PANSS reduction rate) [18, 19]. A 6-week study including adolescents with schizophrenia found that week 3 rather than week 2 improvement (≥ 20% PANSS reduction) was a better predictor of ultimate response to aripiprazole (≥ 40% PANSS reduction) [20].

In this study, we investigate whether early phase treatment response on weeks 2 and 4 could predict therapeutic outcome at week 8 using data from a large multicenter, open-label clinical trial. Secondly, we defined mild, moderate, and severe schizophrenia by PANSS and aimed to investigate the optimum predictive cut-off value defining early non-response in subgroups with different baseline severities of illness. Thirdly, we investigate whether the cut-off value is consistent in different atypical antipsychotic drugs (olanzapine, risperidone, amisulpride, and aripiprazole).

## Methods

### Participants

This study was a multicenter, 8-week, open-label, randomized clinical trial conducted at 19 psychiatric centers throughout China and was registered on Clinicaltrials.gov (NCT03451734). The full trial protocol, including sample size calculation, has been published previously [21]. The study was performed from January 23, 2018, to June 30, 2020. Antipsychotic monotherapy groups were determined using the random number table, and we anticipated that each group would have an equal distribution. Ziprasidone and haloperidol groups mentioned in the registration were canceled in the actual trial owing to the reduced funding compared with original budget. Thus, patients were randomized to receive olanzapine, risperidone, aripiprazole, or amisulpride monotherapy for 8 weeks. Participants were stratified by baseline illness severity. We followed the interpretive guides for the PANSS total scores and linked these scores with the clinical global impression ratings using the anchor-based approach: mild, moderate, and severe schizophrenia have 58–75, 76–95, and no less than 95 on the total score of PANSS respectively [22]. Besides, we followed the Consolidated Standards of Reporting Trials (CONSORT) guidelines to improve the quality of our reporting [23]. The supporting CONSORT checklist is available as supplemental information (Additional file 1: CONSORT checklist).

Participants met the following criteria: (1) aged 18–65 years and meeting the criteria for schizophrenia (diagnosed by the Diagnostic and Statistical Manual of Mental Disorders, Fifth Edition (DSM-5) or International Classification of Diseases, Tenth Edition (ICD-10)); (2) experiencing a current episode of psychotic symptoms with a duration of illness less than 5 years; (3) with at least one guardian that accompanies the patient and supervises patients’ medications; and (4) who signed an informed consent form. The exclusion criteria for participants were as follows: (1) with serious physical illness; (2) with disorders such as alcohol or substance abuse, intellectual disability, or other specific systemic diseases; (3) who are pregnant or breastfeeding; and (4) enrolled in other clinical trials.

### Assessments

Participants were evaluated and followed at four time points after titration (baseline, week 2, week 4, and week 8), and all assessments were performed by psychiatrists trained by a positive and negative syndrome scale (PANSS) institute-certified professor of psychiatry. All the assessors involved in this study received consistency training before the research began. At baseline, sociodemographic information was collected, and disease severity was evaluated using PANSS. Antipsychotics were initially administered at a low dose, gradually adding to the therapeutic dose in 1 week according to the study protocol, and remained the same after titration. Drug dose at 8 weeks was recorded.

### Outcome measures

To examine the ability to predict non-response at the endpoint by the magnitude of PANSS total score improvement at earlier assessment (week 2 or week 4), receiver operating characteristic (ROC) curves with area under the curve (AUC) values were calculated. The predictive values, including total accuracy, sensitivity, specificity, positive predictive value (PPV), and negative predictive value (NPV), were calculated for response status at an earlier assessment (week 2 or week 4) to predict later (week 8) response or non-response, referring to previous literature [10]. In this analysis, sensitivity was defined as the probability that a non-responder would also be rated as not improved at an earlier assessment, and specificity was defined as the probability that a responder would also be rated as improved at an earlier assessment. PPV is defined as the probability that a patient without early improvement showed a subsequent non-response, and NPV is defined as the probability that a patient with early improvement showed a subsequent response. Total accuracy was the proportion of patients whose 2-week or 4-week response status (early response or early non-response) accurately predicted subsequent response status.

Non-response rate to antipsychotic medication, change from baseline to week 8 in PANSS total score, and rate of reduction in PANSS total score after 8 weeks were used for efficacy evaluation. Non-response was defined as a < 20% reduction of the PANSS total score from baseline to week 8. The PANSS total score reduction rate was calculated as (baseline PANSS total score − follow-up PANSS total score)/(baseline PANSS total score − 30) × 100% [24].

### Statistical analysis

The Statistical Package for Social Sciences (version 26.0) was used for statistical analyses. Continuous variables were described using means and standard deviations. Categorical variables were described using frequencies and percentages. The PANSS total score reduction rate, PANSS total score change from baseline, age, and duration among treatment groups were compared by one-way analysis of variance (ANOVA) followed by Bonferroni’s post hoc multiple comparison test. The chi-square or Fisher’s exact test was used to test for differences in the distribution of categorical variables. Further comparisons between groups were performed using the Dunn-Bonferroni post hoc test. Missing values were handled using the last-observation carried-forward method. All statistical tests were two-tailed. Statistical significance was set at *P* < 0.05.

## Results

**Fig. 1:**
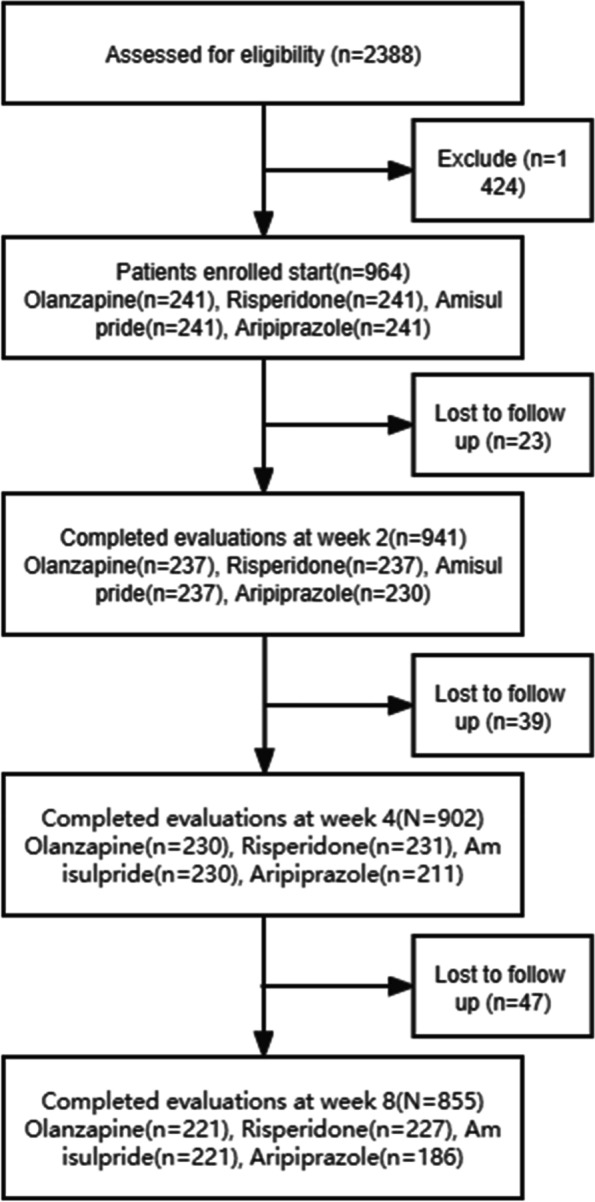
Flowchart of the study design

A total of 2388 patients were admitted and assessed for eligibility, while 1424 were excluded because they did not meet the inclusion criteria or refused to participate. Ultimately, 964 patients were enrolled in the study and randomized to receive olanzapine, risperidone, aripiprazole, or amisulpride monotherapy. Eight hundred and fifty-five of 964 (88.7%) participants completed the 8-week study. The dropout rate of the olanzapine, risperidone, amisulpride, and aripiprazole groups was 8.3%, 5.8%, 8.3%, and 22.8% respectively (see Fig. 1). The mean age and duration of illness were 27.89 ± 8.59 years and 24.81 ± 19.70 months. The average baseline PANSS total score was 84.52 ± 19.20. At the enrollment stage, the numbers of patients with severe, moderate, and mild were 190, 521, and 253, respectively. There were no difference in age, sex, duration, baseline PANSS total scores, and disease severity among the four groups. The average dose was 17.17 ± 4.81 mg/day for olanzapine, 4.55 ± 1.39 mg/day for risperidone, 634.85 ± 198.19 mg/day for amisulpride, and 20.44 ± 6.34 mg/day for aripiprazole. Demographic characteristics are displayed in Table 1.

**Table 1:** Demographic characteristics of the study population

	**Total study population (*n* = 964)**	**Olanzapine (*n* = 241)**	**Risperidone (*n* = 241)**	**Amisulpride (*n* = 241)**	**Aripiprazole (*n* = 241)**
Age (year)	27.89 ± 8.59	28.10 ± 9.79	28.27 ± 8.69	26.94 ± 7.49	28.26 ± 7.49
Sex (male/female)	448/518	116/125	100/141	124/117	108/133
Duration (month)	24.81 ± 19.70	23.19 ± 15.37	24.57 ± 18.35	26.18 ± 20.59	25.31 ± 23.56
PANSS total score	84.52 ± 19.20	85.56 ± 13.75	83.69 ± 13.43	85.03 ± 13.75	83.80 ± 15.96
Dose of antipsychotic (mg/day)		17.17 ± 4.81	4.55 ± 1.39	634.85 ± 198.19	20.44 ± 6.34
Severity of illness					
Severe schizophrenia (PANSS total score > 95)	190	49	42	54	45
Moderate schizophrenia (95 ≥ PANSS total score > 75)	521	133	132	132	124
Mild schizophrenia (75 ≥ PANSS total score > 58)	253	59	67	55	72

Data are presented as means ± standard deviations

*PANSS* Positive and Negative Syndrome Scale

**Fig. 2:**
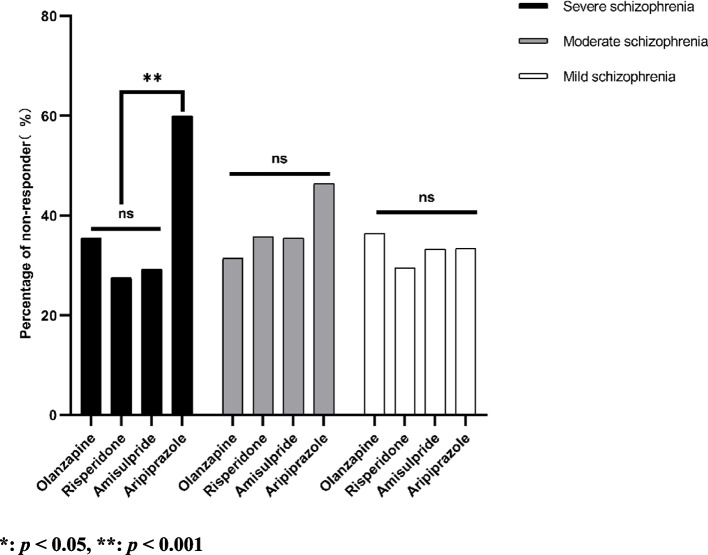
Percentage of non-responder to four antipsychotics at endpoint, stratified by baseline illness severity

We summarized the non-responder rate and the reduction rate of PANSS at week 8, and the percentage of non-responders after 8-week treatment was 35.7%. The non-response rate for olanzapine, risperidone, amisulpride, and aripiprazole were 33.5%, 32.6%, 33.6%, and 44.6%, respectively. The non-response rate of the aripiprazole group was significantly higher than that of the olanzapine, risperidone, and amisulpride groups (*χ*^*2*^ = 8.283,* P* < 0.05). Based on illness severity at baseline, we further compared the percentage of non-response among the four treatment groups (Fig. 2). For patients with severe schizophrenia, the percentage of non-responders was 35.5%, 27.5%, 29.2%, and 60.0% in olanzapine, risperidone, amisulpride, and aripiprazole groups, respectively. The percentage of non-responders was significantly higher in the aripiprazole group than that in the other treatment groups (*P* < 0.001). In patients with mild and moderate schizophrenia, there is no difference among the percentages of non-responders of four groups (moderate schizophrenia: olanzapine 31.4%, risperidone 35.7%, amisulpride 35.5%, and aripiprazole 46.4%; mild schizophrenia: olanzapine 36.4%, risperidone 29.5%, amisulpride 33.3%, and aripiprazole 33.3%).

Table 2 displays the results of an 8-week treatment period for four different groups. The olanzapine group had a decrease of 16.24 ± 11.24 points in PANSS total scores, with a reduction rate of 29.0 ± 16.9%. Similarly, the risperidone group had a decrease of 15.70 ± 10.58 points, with a reduction rate of 29.0 ± 17.2%. The amisulpride group had a decrease of 16.09 ± 10.42 points, with a reduction rate of 29.2 ± 17.0%. The aripiprazole group had a decrease of 12.22 ± 9.40 points, with a reduction rate of PANSS at 23.7 ± 16.4%. Olanzapine, risperidone, and amisulpride caused a greater reduction than aripiprazole in terms of the decrease and reduction rate of PANSS total scores from baseline to week 8 (*P* < 0.01). There was no difference among the olanzapine, risperidone, and amisulpride groups in the decrease and reduction rate of the PANSS total scores from baseline to week 8. We further compared responses to different antipsychotic treatments stratified by baseline illness severity. In patients with moderate and severe schizophrenia, the aripiprazole group also had the least improvement and highest PANSS total scores at week 8 compared with the other three groups (*P* < 0.05). However, no difference was observed in mild schizophrenia in the reduction rate of the PANSS total score.

**Table 2:** The PANSS total score change from baseline and reduction rate of PANSS total score after 8-week treatment, stratified by baseline illness severity

	**Olanzapine group**	**Risperidone group**	**Amisulpride group**	**Aripiprazole group**	**Post-hoc Analysis**					
					**Olanzapine vs risperidone**	**Olanzapine vs amisulpride**	**Olanzapine vs aripiprazole**	**Risperidone vs amisulpride**	**Risperidone vs aripiprazole**	**Amisulpride vs aripiprazole**
**All patients**										
PANSS total score change from baseline	16.24 ± 11.24	15.70 ± 10.58	16.09 ± 10.42	12.22 ± 9.40	0.588	0.882	< 0.001	0.694	0.001	< 0.001
Reduction rate of PANSS total score (%)	29.0 ± 16.9	29.0 ± 17.2	29.2 ± 17.0	23.7 ± 16.4	0.983	0.877	0.002	0.892	0.002	0.001
**Severe schizophrenia** (PANSS total score > 95)										
PANSS total score change from baseline	22.64 ± 16.49	25.05 ± 14.32	22.69 ± 12.58	15.69 ± 12.77	0.432	0.987	0.021	0.435	0.003	0.018
Reduction rate of PANSS total score (%)	29.6 ± 19.6	33.3 ± 18.6	30.9 ± 17.5	19.3 ± 14.9	0.339	0.716	0.006	0.535	< 0.001	0.002
**Moderate schizophrenia** (95 ≥ PANSS total score > 75)										
PANSS total score change from baseline	16.48 ± 9.39	14.87 ± 8.84	15.89 ± 9.69	11.84 ± 9.10	0.173	0.618	0.001	0.388	0.030	0.004
Reduction rate of PANSS total score (%)	30.0 ± 17.2	28.0 ± 17.2	29.6 ± 18.3	23.3 ± 18.4	0.375	0.874	0.013	0.466	0.078	0.018
**Mild schizophrenia** (75 ≥ PANSS total score > 58)										
PANSS total score change from baseline	10.45 ± 5.39	11.28 ± 6.75	10.43 ± 6.25	10.43 ± 6.25	0.459	0.931	0.986	0.415	0.418	0.940
Reduction rate of PANSS total score (%)	26.2 ± 13.2	28.2 ± 16.1	26.6 ± 12.8	27.0 ± 14.3	0.437	0.885	0.793	0.539	0.574	0.918

Data are presented as means ± standard deviations

*PANSS* Positive and Negative Syndrome Scale

In the ROC curve of all the participants, the AUC of week 2 reduction rate for predicting non-response at week 8 was 87.1%, and the AUC of week 4 for predicting non-response at week 8 was 94.5%. For different antipsychotic drugs, the AUC of week 2 for predicting non-response at week 8 was 86.9%, 90.7%, 85.4%, and 84.8% for olanzapine, risperidone, amisulpride, and aripiprazole, respectively. The AUC of week 4 for predicting non-response at week 8 was 95.2%, 94.4%, 93.6%, and 94.9% for olanzapine, risperidone, amisulpride, and aripiprazole. When stratified by baseline illness severity, the AUC of week 2 for predicting non-response at week 8 was 82.9%, 92.1%, and 81.4% for patients with severe, moderate, and mild schizophrenia, respectively. The AUC of week 4 for predicting non-response at week 8 was 94.3%, 94.1%, and 96.0% for patients with severe, moderate, and mild schizophrenia, respectively.

Total accuracy, sensitivity, specificity, and positive and negative predictive values of different cut-off points for predicting non-response at endpoints are shown in Table 3. Higher cut-offs were associated with lower specificity and PPV and higher sensitivity and NPV. Thus, the cut-off of 0% reduction had the highest specificity and PPV, whereas the 20% reduction had the highest sensitivity and NPV. Of notice, the cut-off points of 10% at week 2 and 20% at week 4 had the highest total accuracy (79.2% at week 2 and 90.7% at week 4) comparing the cut-offs ranging from 0 to 20%. When the reduction rate of 10% at week 2 was used as the cut-off value, the sensitivity and PPV were 70.8% and 70.8%, respectively. Using a 20% reduction rate at week 4 as the cut-off value, the sensitivity and PPV were 87.5% and 86.7%, respectively.

**Table 3:** Lack of early improvement cut-offs (0–20%) as predictors of nonresponse to antipsychotics at endpoint

Evaluation time of early improvement	Cutoff value	Total accuracy (%)	Sensitivity (%)	Specificity (%)	PPV (%)	NPV (%)
Week 2	≤ 0%	68.0	12.8	98.7	84.8	67.1
	< 5%	78.6	51.5	93.6	81.7	77.7
	< 10%	79.2	70.8	83.8	70.8	83.8
	< 15%	74.0	93.4	63.2	58.5	94.6
	< 20%	74.0	93.4	63.1	58.5	94.5
Week 4	≤ 0%	64.5	0.7	100.0	100.0	64.4
	< 5%	67.7	8.9	100.0	100.0	66.6
	< 10%	75.6	31.9	99.6	98.0	72.7
	< 15%	85.8	68.0	95.6	89.6	84.4
	< 20%	90.7	87.5	92.5	86.7	93.0

*PPV* Positive predictive value, *NPV* Negative predictive value

The cut-off values vary in different baseline illness severity levels. Additional file 2: Table S1 presented the total accuracy, sensitivity, specificity, PPV, and NPV of various cut-off values in different illness severity levels. We discovered that at week 2, a reduction of < 5% in PANSS showed the highest total accuracy in the severe and mild schizophrenia patients (severe schizophrenia: total accuracy, sensitivity, and PPV of 75.0%, 49.3%, and 76.7%, respectively; mild schizophrenia: total accuracy, sensitivity, and PPV of 80.8%, 55.7%, and 80.0%, respectively). A reduction of < 10% showed the highest total accuracy in the moderate schizophrenia patients (total accuracy, sensitivity, and PPV of 84.0%, 72.8%, and 81.0%, respectively). Index cut-offs ranging from 0 to 20% PANSS reduction at week 4 to predict non-response to antipsychotics at endpoint were also evaluated (Additional file 3: Table S2). At week 4, a reduction in the PANSS total score of 20% was the best predictor in each severity of illness (severe schizophrenia: total accuracy 91.0%, sensitivity 92.6%, and PPV 85.1%; moderate schizophrenia: total accuracy 89.8%, sensitivity 86.1%, and PPV 86.1%; mild schizophrenia: total accuracy 92.1%, sensitivity 86.1%, and PPV 89.5%).

The test characteristics of each cut-off (ranging from 0 to 20%) at week 2 and week 4 in four antipsychotics were shown in Additional file 4: Table S3 and Additional file 5: Table S4. A reduction of < 10% at week 2 showed the highest total accuracy in the olanzapine and aripiprazole groups, with total accuracy, sensitivity, and PPV of 79.2%, 66.2%, and 70.0%, respectively in the olanzapine group, and total accuracy, sensitivity, and PPV of 77.4%, 81.9%, and 71.6%, respectively in the aripiprazole group. A reduction of < 5% at week 2 showed the highest total accuracy in the risperidone and amisulpride groups, with total accuracy, sensitivity, and PPV of 82.4%, 51.4%, and 90.5%, respectively, in the risperidone group, and total accuracy, sensitivity, and PPV of 78.2%, 45.9%, and 81.0%, respectively, in the amisulpride group. A reduction of < 20% at week 4 had the highest total accuracy in the olanzapine, risperidone, amisulpride, and aripiprazole groups, with total accuracy of 90.9%, 90.7%, 90.4%, and 90.8%, respectively.

## Discussion

To investigate the optimum predictive cut-off value defining early non-response at week 2 and week 4 for schizophrenia patients using different atypical antipsychotics (olanzapine, risperidone, amisulpride, and aripiprazole) with different baseline severities of illness (severe, moderate and mild), we evaluated the prediction power under a series reduction of PANSS ranging from 0 to 20%. The main findings were as follows: first, in patients with schizophrenia treated with olanzapine, risperidone, amisulpride, or aripiprazole, those who show poorer early improvement after 2 weeks of antipsychotic treatment were less likely to respond later, and 4 weeks is sufficient to make a definitive determination of ultimate non-responders. Second, overall, the definition of early non-response that < 10% symptom reduction at week 2 and < 20% symptom reduction at week 4 had the best predictive value for non-response at week 8. Third, the optimum predictive cut-off value varies depending on the severity of the psychosis and the atypical antipsychotics being used.

We investigated whether early non-response at week 2 and week 4 could be useful as a marker for assessing later non-response by performing a ROC analysis. The AUC in different subgroups were all ranging from 80 to 95%, indicating moderate diagnostic accuracy to high diagnostic accuracy [25]. Regardless of illness severity and antipsychotic types, our finding indicates symptom reductions at week 2 had acceptable discrimination in predicting non-response, and the response to antipsychotics after week 2 could be taken into consideration in the decision of switching medication. Some previous studies demonstrated the importance of a later time point after 2 weeks in determining early non-response status, supporting our results. In an analysis of first-episode patients, the predictive power of remission was significantly improved after adding assessments of weeks 4 and 6 instead of only including statistics at week 2 [26]. In another study, the percentage decrease in symptom severity score at week 4, but not at week 2, was associated with the response at week 16 [27]. These results are in line with another first-episode study that the time of antipsychotic response varied widely and 22.5% of patients did not respond until they received 4 weeks of treatment [28].

In addition, our study revealed that the optimal prediction thresholds varied with respect to different prediction time points. A < 10% symptom reduction at week 2 and a < 20% symptom reduction at week 4 (early non-response) had the best predictive value for non-response at week 8, which differs from a < 20% symptom reduction at week 2 and a < 30% symptom reduction at week 4 in patients treated with aripiprazole or quetiapine [29]. Maybe somewhat limited by the small sample size, previous studies rarely investigate the optimal cut-off value in different antipsychotic monotherapy group. A previous study enrolled 36 first-episode schizophrenia patients on olanzapine monotherapy and evaluated whether early response (defined as a reduction of 20, 25, and 30% in PANSS score at weeks 2, 3, and 4) could predict a reduction of 50% at week 8, which found 30% in PANSS score at week 4 (with AUC = 92%) can be taken for the prediction [30]. This finding is similar to that of other investigators who have observed a prediction of a reduction of 40% at week 8 by using a reduction of 30% in PANSS score at week 4 [31]. Another study that enrolled 48 first-episode psychosis patients treated with risperidone found that a 20% reduction in PANSS score at week 2 and 30% at week 4 could predict non-responders at week 8 (reduction < 50%) [32]. A possible explanation for these inconsistent results might be various definitions of non-response at endpoint. In our study, a < 20% reduction from baseline to endpoint was applied as non-response to antipsychotics after 8-week treatment, which is an extremely stringent definition of non-response as it reflects “minimal improvement” [33, 34]. One study also defined ultimate non-response as a reduction of < 20% symptoms and found a < 20% reduction at week 2 could predict non-response at week 6 [35]. However, this study used Brief Psychiatric Rating Scale-children (BPRS-C) to assess the severity of symptoms and lacked evaluation of the effects of different cut-offs on the prediction performance.

The optimal cut-off values found in our research are inconsistent with a previous study, in which the optimal cut-off value was < 15% reduction in PANSS total score at week 2 and < 27% at week 4 for moderately-to-severely ill patients, < 12% reduction at week 2 and < 20% at week 4 in less than moderately ill patient group [36]. One possible reason for the above inconsistency may be that the research subjects were different. Our study included schizophrenia patients with an illness duration of 5 years or less, while the study conducted by Chen et al. included chronically ill for a median duration of illness of 16.4 years. Our data partially support the findings of the study by Chen et al. that early non-response cut-off values were generally smaller for patients in the mildly ill group compared to patients in the moderately ill group. A possible explanation for this might be that patients with different baseline severity may experience different trajectories of response to treatment with atypical antipsychotic medication. Stauffer et al. found patients with moderately ill may be expected to have a slow, sustained response to treatment, whereas those who are severely ill may have more promising treatment responses, but with differences in the timing of the response [37].

Our study examined the effects of olanzapine, risperidone, amisulpride, and aripiprazole on patients with early schizophrenia over an 8-week period. We found that all three drugs were equally effective in improving symptoms in patients with severe schizophrenia, while aripiprazole was slightly less effective. In patients with mild schizophrenia, all four drugs showed similar effectiveness during the first 8 weeks of treatment. These results are consistent with previous research [38–40]. However, due to the limited follow-up time of our study, it is challenging to draw clear conclusions about the advantages and disadvantages of drug efficacy. Therefore, longer-term studies are necessary to further compare drug efficacy.

The results of this study should be interpreted taking its limitations into consideration. This study included only four antipsychotics; therefore, further comparisons between drugs were unavailable. Moreover, the endpoint was set at week 8 which limits the prediction of long-term outcomes. Another limitation of our study is the absence of individual comorbidities, including substance use disorder (SUD), which are prevalent in this population. Lastly, we used the full 30-item PANSS to assess early response and non-response, which is a lengthy and unusual measure in clinical practice, potentially limiting the clinical applicability of the findings in real-world settings.

## Conclusions

In conclusion, based on a large multicenter, open-label, randomized trial of 964 patients with schizophrenia receiving monotherapy with olanzapine, risperidone, amisulpride, and aripiprazole, this study confirmed that early non-response to antipsychotics is a predictor of later non-response. Moreover, antipsychotic non-response at week 8 can be predicted as early as week 2, but the optimum predictive cut-offs should be determined based on the antipsychotic type and baseline severity. Moreover, when non-response at week 4 was used as a predictor, the most appropriate predictive cut-off was consistently observed regardless of antipsychotic types or disease severity.

## Supplementary Information

**Additional file 1. **CONSORT checklist. The CONSORT checklist for reporting a randomized trial.

**Additional file 2: Table S1.** Lack of 2 weeks improvement cut-offs as predictors of nonresponse to antipsychotics at endpoint.

**Additional file 3: Table S2.** Lack of 4 weeks improvement cut-offs as predictors of nonresponse to antipsychotics at endpoint.

**Additional file 4: Table S3.** Lack of 2 weeks improvement cut-offs as predictors of nonresponse in four antipsychotics.

**Additional file 5: Table S4.** Lack of 4 weeks improvement cut-offs as predictors of nonresponse in four antipsychotics.

## Data Availability

The datasets used and/or analyzed during the current study are available from the corresponding author on reasonable request.

## Abbreviations

AUC: Area under the curve
CGI-I: Clinical Global Impressions-Improvement Scale
CGI-S: Clinical Global Impression-Severity of Illness Scale
CONSORT: Consolidated Standards of Reporting Trials
NPV: Negative predictive value
PANSS: The positive and negative syndrome scale
PPV: Positive predictive value
ROC: Receiver operating characteristic curves

## References

[1] Samara MT, Nikolakopoulou A, Salanti G, Leucht S (2019). How many patients with schizophrenia do not respond to antipsychotic drugs in the short term? An analysis based on individual patient data from randomized controlled trials. Schizophr Bull.

[2] Kay SR, Fiszbein A, Opler LA (1987). The positive and negative syndrome scale (PANSS) for schizophrenia. Schizophr Bull.

[3] Stauffer VL, Case M, Kinon BJ, Conley R, Ascher-Svanum H, Kollack-Walker S (2011). Early response to antipsychotic therapy as a clinical marker of subsequent response in the treatment of patients with first-episode psychosis. Psychiatry Res.

[4] Hasan A, Falkai P, Wobrock T, Lieberman J, Glenthøj B, Gattaz WF (2017). World Federation of Societies of Biological Psychiatry (WFSBP) guidelines for biological treatment of schizophrenia - a short version for primary care. Int J Psychiatry Clin Pract.

[5] National Institute for Health and Care Excellence (2014). Psychosis and schizophrenia in adults: prevention and management.

[6] Barnes TR, Drake R, Paton C, Cooper SJ, Deakin B, Ferrier IN (2020). Evidence-based guidelines for the pharmacological treatment of schizophrenia: updated recommendations from the British Association for Psychopharmacology. J Psychopharmacol.

[7] Lin CH, Lin HS, Lin SC, Kuo CC, Wang FC, Huang YH (2018). Early improvement in PANSS-30, PANSS-8, and PANSS-6 scores predicts ultimate response and remission during acute treatment of schizophrenia. Acta Psychiatr Scand.

[8] Chen YL, Chen KP, Chiu CC, Tai MH, Lung FW (2018). Early predictors of poor treatment response in patients with schizophrenia treated with atypical antipsychotics. BMC Psychiatry.

[9] Ye W, Montgomery W, Kadziola Z, Liu L, Xue H, Stensland MD (2014). Factors associated with early response to olanzapine and clinical and functional outcomes of early responders treated for schizophrenia in the People's Republic of China. Neuropsychiatr Dis Treat.

[10] Samara MT, Leucht C, Leeflang MM, Anghelescu IG, Chung YC, Crespo-Facorro B (2015). Early improvement as a predictor of later response to antipsychotics in schizophrenia: a diagnostic test review. Am J Psychiatry.

[11] Furukawa TA, Levine SZ, Tanaka S, Goldberg Y, Samara M, Davis JM (2015). Initial severity of schizophrenia and efficacy of antipsychotics: participant-level meta-analysis of 6 placebo-controlled studies. JAMA Psychiat.

[12] Rabinowitz J, Werbeloff N, Caers I, Mandel FS, Stauffer V, Menard F (2014). Determinants of antipsychotic response in schizophrenia: implications for practice and future clinical trials. J Clin Psychiatry.

[13] Stern RG, Kahn RS, Davidson M (1993). Predictors of response to neuroleptic treatment in schizophrenia. Psychiatr Clin North Am.

[14] Lambert M, Schimmelmann BG, Naber D, Eich FX, Schulz H, Huber CG (2009). Early- and delayed antipsychotic response and prediction of outcome in 528 severely impaired patients with schizophrenia treated with amisulpride. Pharmacopsychiatry.

[15] Kinon BJ, Chen L, Ascher-Svanum H, Stauffer VL, Kollack-Walker S, Sniadecki JL (2008). Predicting response to atypical antipsychotics based on early response in the treatment of schizophrenia. Schizophr Res.

[16] Hatta K, Otachi T, Sudo Y, Hayakawa T, Ashizawa Y, Takebayashi H (2011). Difference in early prediction of antipsychotic non-response between risperidone and olanzapine in the treatment of acute-phase schizophrenia. Schizophr Res.

[17] Chang YC, Lane HY, Yang KH, Huang CL (2006). Optimizing early prediction for antipsychotic response in schizophrenia. J Clin Psychopharmacol.

[18] Rasmussen SA, Rosebush PI, Anglin RE, Mazurek MF (2016). The predictive value of early treatment response in antipsychotic-naive patients with first-episode psychosis: Haloperidol versus olanzapine. Psychiatry Res.

[19] Rasmussen SA, Rosebush PI, Mazurek MF (2017). Does early antipsychotic response predict long-term treatment outcome?. Hum Psychopharmacol.

[20] Correll CU, Zhao J, Carson W, Marcus R, McQuade R, Forbes RA (2013). Early antipsychotic response to aripiprazole in adolescents with schizophrenia: predictive value for clinical outcomes. J Am Acad Child Adolesc Psychiatry.

[21] Xiao J, Huang J, Long Y, Wang X, Wang Y, Yang Y (2021). Optimizing and individualizing the pharmacological treatment of first-episode schizophrenic patients: study protocol for a multicenter clinical trial. Front Psychiatry.

[22] Leucht S, Kane JM, Kissling W, Hamann J, Etschel E, Engel RR (2005). What does the PANSS mean?. Schizophr Res.

[23] Schulz KF, Altman DG, Moher D (2010). CONSORT 2010 statement: updated guidelines for reporting parallel group randomised trials. BMJ.

[24] Leucht S, Davis JM, Engel RR, Kissling W, Kane JM (2009). Definitions of response and remission in schizophrenia: recommendations for their use and their presentation. Acta Psychiatr Scand Suppl.

[25] Swets JA (1988). Measuring the accuracy of diagnostic systems. Science.

[26] Andreasen NC, Carpenter WT, Kane JM, Lasser RA, Marder SR, Weinberger DR (2005). Remission in schizophrenia: proposed criteria and rationale for consensus. Am J Psychiatry.

[27] Gallego JA, Robinson DG, Sevy SM, Napolitano B, McCormack J, Lesser ML (2011). Time to treatment response in first-episode schizophrenia: should acute treatment trials last several months?. J Clin Psychiatry.

[28] Emsley R, Rabinowitz J, Medori R (2006). Time course for antipsychotic treatment response in first-episode schizophrenia. Am J Psychiatry.

[29] Pagsberg AK, Krogmann A, Jeppesen P, von Hardenberg L, Klauber DG, Jensen KG (2022). Early antipsychotic nonresponse as a predictor of nonresponse and nonremission in adolescents with psychosis treated with aripiprazole or quetiapine: results from the TEA trial. J Am Acad Child Adolesc Psychiatry.

[30] Subeesh V, Maheswari E, Singh H, Saraswathy GR, Reddy N, Chiranjeevi P (2020). Early prediction of clinical response in first episode schizophrenia (FES) patients receiving olanzapine. Int J Psychiatry Clin Pract.

[31] Ascher-Svanum H, Zhao F, Detke HC, Nyhuis AW, Lawson AH, Stauffer VL (2011). Early response predicts subsequent response to olanzapine long-acting injection in a randomized, double-blind clinical trial of treatment for schizophrenia. BMC Psychiatry.

[32] Subeesh V, Maheswari E, Singh H, Neha R, Mazhar F (2021). Finding early improvement threshold to predict response after 8 weeks of treatment using risperidone in first-episode psychosis. J Clin Psychopharmacol.

[33] Levine SZ, Rabinowitz J, Engel R, Etschel E, Leucht S (2008). Extrapolation between measures of symptom severity and change: an examination of the PANSS and CGI. Schizophr Res.

[34] Leucht S, Engel RR, Davis JM, Kissling W, Meyer Zur Capellen K, Schmauß M (2012). Equipercentile linking of the Brief Psychiatric Rating Scale and the Clinical Global Impression Scale in a catchment area. Eur Neuropsychopharmacol.

[35] Stentebjerg-Olesen M, Ganocy SJ, Findling RL, Chang K, DelBello MP, Kane JM (2015). Early response or nonresponse at week 2 and week 3 predict ultimate response or nonresponse in adolescents with schizophrenia treated with olanzapine: results from a 6-week randomized, placebo-controlled trial. Eur Child Adolesc Psychiatry.

[36] Chen L, Ascher-Svanum H, Stauffer V, Kinon BJ, Kollack-Walker S, Ruberg S (2009). Optimal thresholds of early response to atypical antipsychotics: application of signal detection methods. Schizophr Res.

[37] Stauffer V, Case M, Kollack-Walker S, Ascher-Svanum H, Ball T, Kapur S (2011). Trajectories of response to treatment with atypical antipsychotic medication in patients with schizophrenia pooled from 6 double-blind, randomized clinical trials. Schizophr Res.

[38] Fleischhacker WW, McQuade RD, Marcus RN, Archibald D, Swanink R, Carson WH (2009). A double-blind, randomized comparative study of aripiprazole and olanzapine in patients with schizophrenia. Biol Psychiatry.

[39] Kishi T, Matsuda Y, Matsunaga S, Iwata N (2015). Aripiprazole for the management of schizophrenia in the Japanese population: a systematic review and meta-analysis of randomized controlled trials. Neuropsychiatr Dis Treat.

[40] Johnsen E, Kroken RA, Løberg E-M, Rettenbacher M, Joa I, Larsen TK (2020). Amisulpride, aripiprazole, and olanzapine in patients with schizophrenia-spectrum disorders (BeSt InTro): a pragmatic, rater-blind, semi-randomised trial. Lancet Psychiatry.

