# Supplementary material for: Early non-response as a predictor of later non-response to antipsychotics in schizophrenia: a randomized trial
Source: BMC Med. 2023 Jul 19;21:263. doi: 10.1186/s12916-023-02968-7 (PMC10354903; doi:10.1186/s12916-023-02968-7)
Supplement: Supplementary file 2 — Additional file 2: Table S1. Lack of 2 weeks improvement cut-offs as predictors of nonresponse to antipsychotics at endpoint. [file 12916_2023_2968_MOESM2_ESM.docx]

**Table S1** Lack of 2 weeks improvement cut-offs as predictors of nonresponse to antipsychotics at endpoint

|  | **Cut-off value** | **Total accuracy (%)** | **Sensitivity (%)** | **Specificity (%)** | **PPV (%)** | **NPV (%)** |
| --- | --- | --- | --- | --- | --- | --- |
| **Severe schizophrenia** |  |  |  |  |  |  |
|  | ≤0% | 66.3 | 13.2 | 99.1 | 90.0 | 64.9 |
|  | <5% | 75.0 | 49.3 | 90.8 | 76.7 | 74.4 |
|  | <10% | 72.5 | 67.7 | 75.5 | 63.0 | 79.0 |
|  | <15% | 71.9 | 91.2 | 60.0 | 58.5 | 91.7 |
|  | <20% | 71.9 | 91.2 | 60.0 | 58.5 | 91.7 |
| **Moderate schizophrenia** |  |  |  |  |  |  |
|  | ≤0% | 67.3 | 11.4 | 98.9 | 85.7 | 66.3 |
|  | <5% | 78.9 | 50.3 | 95.0 | 84.9 | 77.2 |
|  | <10% | 84.0 | 72.8 | 90.3 | 81.0 | 85.4 |
|  | <15% | 80.3 | 97.5 | 70.6 | 65.3 | 98.0 |
|  | <20% | 80.3 | 97.5 | 70.6 | 65.3 | 98.0 |
| **Mild schizophrenia** |  |  |  |  |  |  |
|  | ≤0% | 70.7 | 15.2 | 98.1 | 80.0 | 70.1 |
|  | <5% | 80.8 | 55.7 | 93.1 | 80.0 | 81.0 |
|  | <10% | 75.3 | 69.6 | 78.1 | 61.1 | 83.9 |
|  | <15% | 64.0 | 87.3 | 52.5 | 47.6 | 89.4 |
|  | <20% | 63.9 | 87.3 | 52.2 | 47.6 | 89.2 |

*PPV* positive predictive value, *NPV* negative predictive value
