# Supplementary material for: Early non-response as a predictor of later non-response to antipsychotics in schizophrenia: a randomized trial
Source: BMC Med. 2023 Jul 19;21:263. doi: 10.1186/s12916-023-02968-7 (PMC10354903; doi:10.1186/s12916-023-02968-7)
Supplement: Supplementary file 3 — Additional file 3: Table S2. Lack of 4 weeks improvement cut-offs as predictors of nonresponse to antipsychotics at endpoint. [file 12916_2023_2968_MOESM3_ESM.docx]

**Table S2** Lack of 4 weeks improvement cut-offs as predictors of nonresponse to antipsychotics at endpoint

|  | **Cut-off value** | **Total accuracy (%)** | **Sensitivity (%)** | **Specificity (%)** | **PPV (%)** | **NPV (%)** |
| --- | --- | --- | --- | --- | --- | --- |
| **Severe schizophrenia** |  |  |  |  |  |  |
|  | ≤0% | 62.4 | 1.5 | 100.0 | 100.0 | 62.1 |
|  | <5% | 65.2 | 8.8 | 100.0 | 100.0 | 64.0 |
|  | <10% | 74.0 | 32.8 | 99.1 | 95.7 | 70.8 |
|  | <15% | 85.4 | 69.1 | 95.5 | 90.4 | 83.3 |
|  | <20% | 91.0 | 92.6 | 90.0 | 85.1 | 95.2 |
| **Moderate schizophrenia** |  |  |  |  |  |  |
|  | ≤0% | 64.1 | 0.6 | 100.0 | 100.0 | 64.0 |
|  | <5% | 67.1 | 8.3 | 100.0 | 100.0 | 66.1 |
|  | <10% | 76.3 | 34.2 | 99.6 | 98.1 | 73.2 |
|  | <15% | 87.2 | 73.2 | 95.0 | 89.1 | 86.3 |
|  | <20% | 89.8 | 86.1 | 92.0 | 86.1 | 92.0 |
| **Mild schizophrenia** |  |  |  |  |  |  |
|  | ≤0% | 66.9 | 0.0 | 100.0 | .. | 66.9 |
|  | <5% | 70.6 | 10.3 | 100.0 | 100.0 | 69.6 |
|  | <10% | 75.7 | 26.6 | 100.0 | 100.0 | 73.4 |
|  | <15% | 83.5 | 56.4 | 96.9 | 89.8 | 81.9 |
|  | <20% | 92.1 | 86.1 | 95.0 | 89.5 | 93.3 |

*PPV* positive predictive value, *NPV* negative predictive value
