# Supplementary material for: Early non-response as a predictor of later non-response to antipsychotics in schizophrenia: a randomized trial
Source: BMC Med. 2023 Jul 19;21:263. doi: 10.1186/s12916-023-02968-7 (PMC10354903; doi:10.1186/s12916-023-02968-7)
Supplement: Supplementary file 4 — Additional file 4: Table S3. Lack of 2 weeks improvement cut-offs as predictors of nonresponse in four antipsychotics. [file 12916_2023_2968_MOESM4_ESM.docx]

**Table S3** Lack of 2 weeks improvement cut-offs as predictors of nonresponse in four antipsychotics

|  | **Cut-off value** | **Total accuracy (%)** | **Sensitivity (%)** | **Specificity (%)** | **PPV (%)** | **NPV (%)** |
| --- | --- | --- | --- | --- | --- | --- |
| **Olanzapine** |  |  |  |  |  |  |
|  | ≤0% | 66.5 | 0.0 | 100.0 | .. | 66.5 |
|  | <5% | 77.9 | 40.3 | 96.6 | 85.3 | 76.5 |
|  | <10% | 79.2 | 66.2 | 85.7 | 70.0 | 83.4 |
|  | <15% | 77.8 | 91.9 | 70.7 | 61.3 | 94.5 |
|  | <20% | 77.7 | 91.9 | 70.5 | 61.3 | 94.5 |
| **Risperidone** |  |  |  |  |  |  |
|  | ≤0% | 67.8 | 1.4 | 100.0 | 100.0 | 67.7 |
|  | <5% | 82.4 | 51.4 | 97.4 | 90.5 | 80.5 |
|  | <10% | 81.9 | 71.6 | 86.9 | 72.6 | 86.4 |
|  | <15% | 74.9 | 91.9 | 66.7 | 57.1 | 94.4 |
|  | <20% | 74.9 | 91.9 | 66.7 | 57.1 | 94.4 |
| **Amisulpride** |  |  |  |  |  |  |
|  | ≤0% | 67.3 | 2.7 | 100.0 | 100.0 | 67.0 |
|  | <5% | 78.2 | 45.9 | 94.5 | 81.0 | 77.5 |
|  | <10% | 77.7 | 62.2 | 85.6 | 68.7 | 81.7 |
|  | <15% | 76.4 | 91.9 | 68.5 | 59.6 | 94.3 |
|  | <20% | 76.4 | 91.9 | 68.5 | 59.6 | 94.3 |
| **Aripiprazole** |  |  |  |  |  |  |
|  | ≤0% | 71.0 | 43.4 | 93.2 | 83.7 | 67.1 |
|  | <5% | 75.3 | 66.3 | 82.5 | 75.3 | 75.2 |
|  | <10% | 77.4 | 81.9 | 73.8 | 71.6 | 83.5 |
|  | <15% | 65.6 | 97.6 | 39.8 | 56.6 | 95.3 |
|  | <20% | 65.6 | 97.6 | 39.8 | 56.6 | 95.3 |

*PPV* positive predictive value, *NPV* negative predictive value
