# Supplementary material for: Early non-response as a predictor of later non-response to antipsychotics in schizophrenia: a randomized trial
Source: BMC Med. 2023 Jul 19;21:263. doi: 10.1186/s12916-023-02968-7 (PMC10354903; doi:10.1186/s12916-023-02968-7)
Supplement: Supplementary file 5 — Additional file 5: Table S4. Lack of 4 weeks improvement cut-offs as predictors of nonresponse in four antipsychotics. [file 12916_2023_2968_MOESM5_ESM.docx]

**Table S4** Lack of 4 weeks improvement cut-offs as predictors of nonresponse in four antipsychotics

|  | **Cut-off value** | **Total accuracy (%)** | **Sensitivity (%)** | **Specificity (%)** | **PPV (%)** | **NPV (%)** |
| --- | --- | --- | --- | --- | --- | --- |
| **Olanzapine** |  |  |  |  |  |  |
|  | ≤0% | 66.5 | 0.0 | 100.0 | .. | 66.5 |
|  | <5% | 67.7 | 2.7 | 100.0 | 100.0 | 67.4 |
|  | <10% | 73.2 | 19.2 | 100.0 | 100.0 | 71.4 |
|  | <15% | 86.2 | 63.9 | 97.3 | 92.0 | 84.5 |
|  | <20% | 90.9 | 86.5 | 93.2 | 86.5 | 93.2 |
| **Risperidone** |  |  |  |  |  |  |
|  | ≤0% | 67.4 | 0.0 | 100.0 | .. | 67.4 |
|  | <5% | 69.2 | 5.4 | 100.0 | 100.0 | 68.6 |
|  | <10% | 78.0 | 32.4 | 100.0 | 100.0 | 75.4 |
|  | <15% | 88.5 | 73.0 | 96.1 | 90.0 | 88.0 |
|  | <20% | 90.7 | 85.1 | 93.5 | 86.3 | 92.9 |
| **Amisulpride** |  |  |  |  |  |  |
|  | ≤0% | 66.4 | 0.0 | 100.0 | .. | 66.4 |
|  | <5% | 67.9 | 2.8 | 100.0 | 100.0 | 67.6 |
|  | <10% | 75.1 | 23.9 | 100.0 | 100.0 | 73.0 |
|  | <15% | 84.5 | 62.2 | 95.9 | 88.5 | 83.3 |
|  | <20% | 90.4 | 83.8 | 93.8 | 87.3 | 91.8 |
| **Aripiprazole** |  |  |  |  |  |  |
|  | ≤0% | 56.5 | 2.4 | 100.0 | 100.0 | 56.0 |
|  | <5% | 65.6 | 22.9 | 100.0 | 100.0 | 61.7 |
|  | <10% | 76.3 | 49.4 | 98.1 | 95.3 | 70.6 |
|  | <15% | 83.3 | 72.3 | 92.2 | 88.2 | 80.5 |
|  | <20% | 90.8 | 94.0 | 88.2 | 86.7 | 94.7 |

*PPV* positive predictive value, *NPV* negative predictive value
